# Supplementary figures and images for: Mechanical loading of cranial joints minimizes the craniofacial phenotype in Crouzon syndrome
Source: Sci Rep. 2022 Jun 11;12:9693. doi: 10.1038/s41598-022-13807-9 (PMC9188582; doi:10.1038/s41598-022-13807-9)

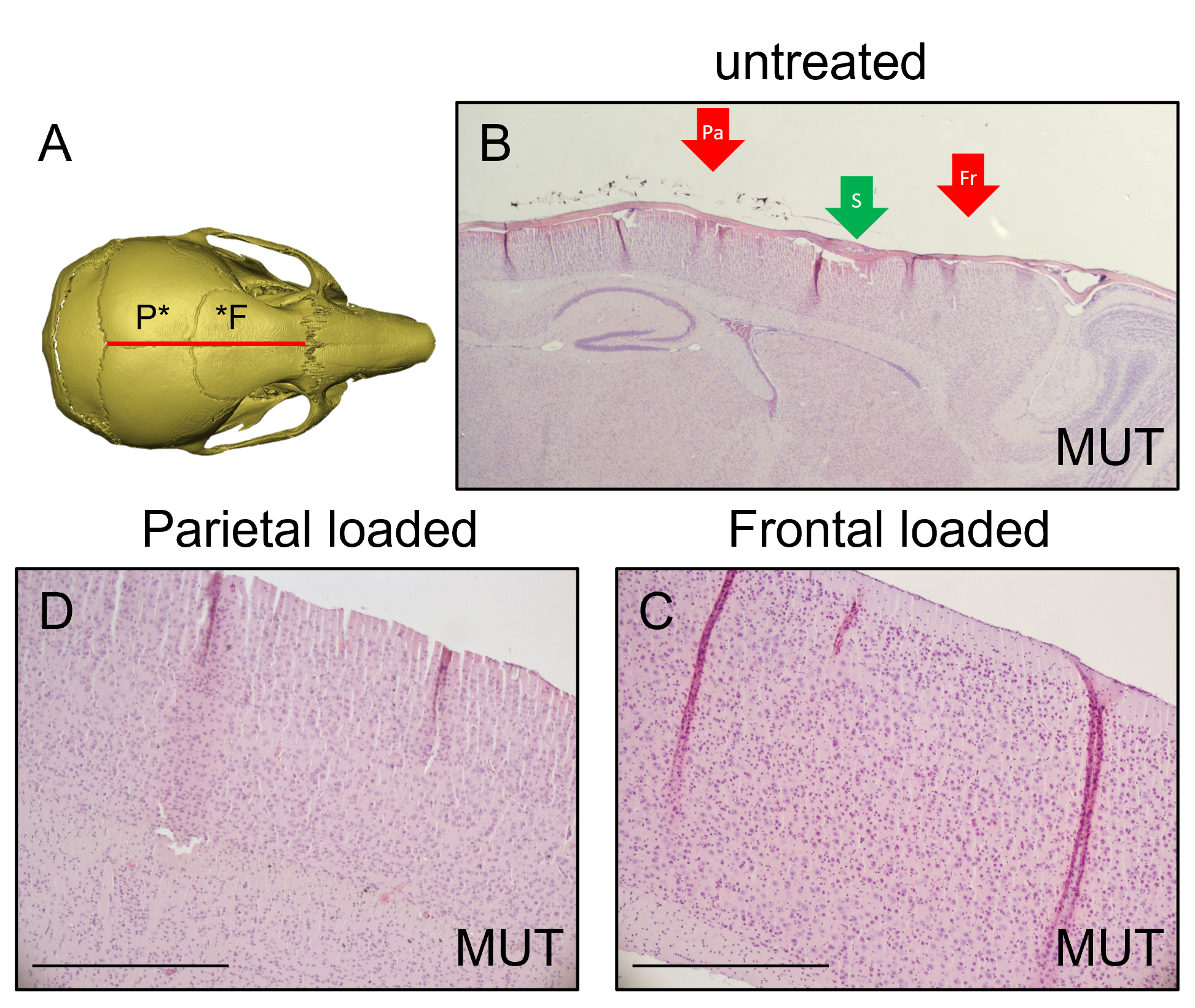

Supplement: Supplementary file 1 — Supplementary Figure 1. [file 41598_2022_13807_MOESM1_ESM.tif]
