# Supplementary material for: Mechanical loading of cranial joints minimizes the craniofacial phenotype in Crouzon syndrome
Source: Sci Rep. 2022 Jun 11;12:9693. doi: 10.1038/s41598-022-13807-9 (PMC9188582; doi:10.1038/s41598-022-13807-9)
Supplement: Supplementary file 2 — Supplementary Legends. [file 41598_2022_13807_MOESM2_ESM.docx]

**Supplementary figure legends**

**Supplementary Figure 1** **Histological analysis of brain tissue following cranial bone loading**. **A** Sagittal, midline sections through the brain (red line) at P21 were stained with H&E. **B** Low magnification image of a mutant brain showing the fused coronal suture (green arrow, S) as well as the approximate location of frontal (red arrow, Fr) and parietal (red arrow, Pa) loading. **C** High magnification image of a frontal loaded mutant brain showing normal cortical lamination. **D** High magnification image of a parietal loaded mutant brain showing normal cortical lamination.

**Supplementary Video 1a** Video footage (25s) of cranial bone loading treatment protocol. This video shows a P7 WT mouse being loaded on the frontal bone.

**Supplementary Video 1b** Video footage (25s) of cranial bone loading treatment protocol. This video shows a P14 WT mouse being loaded on the frontal bone.

**Supplementary Video 2a** Video footage (52s) showing behaviour of three WT P21 litter mates (L19, L20 and L21) after completing the two-week cranial bone loading treatment protocol.

**Supplementary Video 2b** Video footage showing behaviour of three MUT P21 litter mates (L18, L25 and L26) after completing the two-week cranial bone loading treatment protocol.
